# Supplementary material for: Mother-infant interaction characteristics associate with infant falling reactivity and child peer problems at pre-school age
Source: PLoS One. 2024 Jun 4;19(6):e0302661. doi: 10.1371/journal.pone.0302661 (PMC11149888; doi:10.1371/journal.pone.0302661)
Supplement: S1 File — (DOCX) [file pone.0302661.s001.docx]

**Mother-infant interaction characteristics associate with infant falling reactivity and child peer problems at pre-school age**

**Supporting Information**

**Interaction Behaviours Coding Scheme**

We employed a coding scheme that comprised six codes, two relating to maternal behaviours, two to infant behaviours, and two to dyadic interaction behaviours. The detailed coding guide specifically adapted for this study with examples for each code and each Likert scale item from 1 to 7 can be found below.

I. Parent Rating Scales

1. Maternal Supportive Presence^A,C,E^
2. Maternal Respect for Infant's Autonomy^A,C,E^

II. Infant Rating Scales

1. Infant Agency, Autonomy^A,C,E^
2. Infant Negativity^A,C^

III. Dyadic Interaction Scales

1. Dyadic Affective Mutuality^B,C^
2. Dyadic Reciprocity^E^

Adapted from:

^A^Egeland, B. & Hiester, M. (1993). Teaching task rating scales. Institute of Infant Development.

^B^Pianta, R.C. (1994). Rating scales for parent-infant interaction in preschoolers. University of Virginia.

^C^ Owen, M.T., Vaughn, A., Barfoot, B. & Ware, A. (1996). The NICHD Study of Early Infant Care Parent Infant Interaction Rating Scales: Early Infanthood. University of Texas at Dallas.

^D^Hirschmann, N (2013). INTAKT. University of Vienna.

^E^Deater-Deckard, K., Pylas, M.V., Petrill, S.A. (1997). PARCHISY: Parent-Infant Interaction System. University of London.

1. **Parent rating scales**
2. Maternal Supportive Presence (MSP)

A parent scoring high on this scale expresses positive regard and emotional support to the infant. This may occur by acknowledging the infant’s accomplishments on the task / game he/she is doing (e.g., building a house of blocks), encouraging the infant with positive emotional regard (e.g., “You're really good at this.” “You got another one right.”) and various other ways of letting the infant know that he/she has his/her support and confidence to do well in the setting (scaffolding, open-ended questions, explanations). If the infant is having difficulty on the task / game, the parent is reassuring and calm, providing an affectively positive “secure base” for them, perhaps leaning closer to give a physical sense of support.

A parent scoring low on this scale fails to provide supportive cues; he/she might be passive, uninvolved, aloof, or otherwise unavailable to the infant. He or she might also criticize the infant. The parent might also give observers the impression that he/she is more concerned about his/her own adequacy in the setting rather than concerned about the infant’s emotional needs. A potential difficulty in scoring this scale is the need to discount messages of parents that are seemingly supportive in verbal content but are contradicted by other aspects of the communication, e.g., the parent seems to be performing a supportive role for the camera but is not really engaged in what the infant is doing or feeling. Signs of such questionable support are improper timing of support, mismatch of verbal and bodily cues, and failure to have the infant’s attention in delivering the message. These types of supportive messages would not be weighted highly because such features suggest that supportive presence is not a well-practiced aspect of their interaction outside the laboratory setting. Conversely, a parent may be more supportive than he/she appears in this situation because he/she has approached this task as a test of the infant’s achievement and has not used as much support as he/she otherwise might have. Yet, the qualitative features of his/her support would merit a high score.

1. Very Low. Parent completely fails to be supportive to the infant, either being aloof and unavailable or being hostile toward the infant when the infant shows need of some support. Reliance on critical comments (“no, don’t do that”).
2. Low. Parent provides very little emotional support to the infant, or does that only after the infant has become upset.
3. Moderately Low. Parent gives some support but it is sporadic and poorly timed to the infant’s needs. The consistency of this support is uneven so as to make the parent unreliable as a supportive presence.
4. Moderate. This parent does a respectable job of being available when his/her infant needs support. He/she may lean closer as the infant shows small signs of frustration and praise the infant’s efforts to show that he/she is available and supportive, but inconsistency in this style makes his/her support unreliable or unavailable at crucial times in the session.
5. Moderately High. Parent provides good support, reassurance and confidence in the infant’s ability, but he/she falters in this at times when the infant especially could use more support. Or, parent is universally supportive but rarely gives evidence of modulation to the infant’s needs.
6. High. Parent establishes him/herself as supportive and encouraging toward the infant and continues to provide support when the infant needs it. If the infant experiences more difficulty, his/her support increases in commensurate fashion. He/she has some lapses, however, in which the infant’s involvement with the play materials wavers for lack of support. Yet, he/she then attempts to return the infant to a level of involvement that is more optimal.
7. Very High. Parent skilfully provides support throughout the session. He/she sets up the situation from the beginning as one in which he/she is confident of the infant’s efforts. He/she may redirect the infant when appropriate in a way that does not reduce his/her support and confidence in the infant's ability to modify his or her behaviour. If the infant is having difficulty, he/she finds ways to reward some sort of success by the infant and encourage whatever solution the infant can make. Parent is not only emotionally supportive but also continuously reinforces the infant's success.
8. Maternal Respect for Infant’s Autonomy (MRIA)

This scale reflects the degree to which the parent acted in a way that recognized and respected the validity of the infant’s individuality, motives, and perspectives in the session. A parent scoring low on this scale would be very intrusive in his/her interventions with the infant, exerting his/her expectations on the infant in a way that makes the infant a satellite or servant of the parent rather than a partner in a mutually negotiated relationship. Or the parent might implicitly define his/her interactions in terms of a win-lose power struggle in which compliance by the infant makes parent the winner and the infant submissive. Parents may intrude either harshly or with affection; in either case, his/her actions do not acknowledge the infant’s intentions as real or valid and communicate that it is better and safer to depend on him/her for direction than to attempt individuality. The parent might take over the task/game the infant is engaged with and solve it by himself or herself. In contrast, a parent scoring high on this scale acknowledges the infant’s perspectives and desires as a valid part of the infant’s individual identity. A parent scoring very high does this explicitly by negotiating rules with the infant, verbalizing his/her acknowledgement of the infant's intentions, does not deny the infant’s right to those desires, and models his/her own identity and the validity of his/her own desires in the way he/she expects the infant to respect his/her individuality, too. Parent can get a low score just by denying the infant’s individuality strongly (e.g., doing things before the infant can on his/her own, etc.) even though it is not interrupting the infant’s behaviour.

1. Very low. Parent completely denies the infant’s individuality in the techniques he/she uses. Parent is very intrusive, physical and forceful in controlling the infant.
2. Low. Parent strongly denies the infant’s individuality, but there are a few opportunities for the infant to experience autonomy, whether by variation in parent’s approach or simply by occasional absence of maternal controls over the infant. Mostly, however, this parent’s style denies the infant’s autonomy and is intrusive. Mostly explicit directions.
3. Moderately low. Parent does not completely deny the infant’s individuality, but he/she effectively communicates that the infant’s intentions do not have validity compared to his/her own intentions for the infant. He/she also intrudes strongly on the infant’s behaviour, giving him/her little chance to do anything on his or her own. Reliance on explicit directions (“up, down, stop”).
4. Moderate. Parent shows moderate respect for infant’s autonomy. He/she is moderately intrusive. Although parent does not deny the infant’s separate identity, he/she does very little to support the validity of the infant’s individuality. He/she might communicate doubts to the infant about the appropriateness of having his/her own intentions, or intrude abruptly on the infant several times. Reliance on explicit directions (“up, down, stop”), but also moments of praise and encouragement.
5. Moderately high. Parent does allow the infant some autonomy of intentions, but he/she does not actively support and reinforce this perspective in the infant. He/she may reflect the infant’s intentions and ideas by engaging the infant, but he/she also exerts his/her will at times over the infant in a way that shifts the infant’s perspective. This rating is also given when the parent, by lack of involvement, does not give the infant an opportunity to experience autonomy.
6. High. Parent respects infant’s autonomy. He/she is not intrusive over the infant; instead, he/she acknowledges the infant’s intentions, communicates trust in the infant’s individuality, and allows a mutually negotiated interaction.
7. Very high. Parent very clearly interacts with the infant in a way that acknowledges the validity of the infant’s perspective, encourages the infant to acknowledge his/her intentions, and to negotiate the course of interactions in the session. This parent also models his/her individuality to the infant in these negotiated interactions and may insist on the importance of his/her interventions being followed, but he/she does so while acknowledging the reality and validity of the infant’s differing perspective and never in an intrusive manner.
8. **Infant rating scales**
9. Infant’s Agency/Autonomy (IA)

The infant acts with vigour, confidence, and eagerness to play / do the tasks. The infant takes an active interest in his/her activities, invests effort in them (although not necessarily very persistent), and appreciates successes. Agency includes a sense of coordination between affect and behaviour. Infant should appear well integrated in the sense of directing his/her energy into activities without conflicting motivations or repression of feelings and with confidence that everything is okay. Agency must be scored for goal-oriented behaviour on the tasks / games. Other goals or expressions of excitement may be in service of distracting the parent, winning approval, etc., and would not represent agency here.

1. Very low. Infant displays no agency, seems hesitant to engage problems or does so “mechanically” and with no evidence of being interested in or excited by his/her performance (although this infant may nonetheless be distraught over failures). Infant shows extreme lack of confidence in his/her behaviour and is affectively restrained. Mother leads throughout the tasks / games.
2. Low. Infant generally does not display agency. Infant does take some active interest in his/her activities, shows some enthusiasm and becomes engaged for brief periods, but is mostly restrained. Mostly mother leads the tasks / games.
3. Moderately low. Infant shows some clear moments of agency and active, enthusiastic engagement in her/his activities but primarily she/he does not engage with the situation in this way. Mostly mother leads the task / game and infant engagement changes quickly.
4. Moderate. Infant shows a mixture of enthusiasm and restraint or superficiality of effort. This may occur because the infant is very slow in “warming up” to the potential of the situation or because his/her enthusiasm waxes and wanes and he or she is not reliably invested in the activities.
5. Moderately high. The infant displays agency for much of the session and is basically interested in and enthused about his/her activities. There is a sense of harmony between affect and behaviour in the infant’s enthusiasm, but infant also has periods in which this is not the case. The infant is leading the task / game for about half of the time.
6. High. Infant demonstrates agency, enthusiasm and coordinated affect and behaviour for most of the session with only brief and minor periods in which this is not so. The infant is quite eager and confident in approaching the activities and enjoys her/his accomplishments. The infant is mostly leading the task / game and there are one or two instances of following the mother’s lead.
7. Very high. Infant shows high agency and enthusiasm in activities throughout the session. Infant approaches goals eagerly, and with some persistence when she/he encounters difficulties, and the coordination of affect with behaviour gives the infant a notable sense of energy in all activities. Infant clearly “jumps” on tasks / games with eagerness and wants to get involved. The infant controls the task / game from beginning to end.
8. Infant Negativity (IN)

Infant negativity is the degree to which the infant shows anger, dislike, or hostility toward the parent. At the high end, the infant is repeatedly and overtly angry at the parent, e.g., forcefully rejecting his/her ideas, showing angry and resistant expression, pouting, or being unreasonably demanding or critical of him/her. For the lowest rating, there are neither overt nor covert signs of such anger. Expressions are essentially positive toward parent whether or not the infant is compliant or much involved with him/her. Low ratings may include brief instances of frustration or rejections of parent’s help. Failure to answer the parent’s questions without signs of deliberate ignoring is not to be considered negativity.

1. Very Low. Infant shows no signs of negativism. She/he shows through consistently positive interactions toward the parent that s/he has a truly positive relationship toward him/her and feels no abiding anger toward him/her.
2. Low. Infant shows no clear indications of negativism, but the tone of some interactions is less positive than one would desire in an ideal relationship toward the parent.
3. Moderately Low. Infant is negativistic only briefly in any overt fashion, but these suggest some noticeable anger and resistance in the infant’s interactions with parent.
4. Moderate. Infant shows clear negativism toward the parent on several occasions or one significant occasion, but these are rather isolated episodes.
5. Moderately High. Infant is frequently negativistic or a few instances of strong or intense negativism, but these are not predominant in the interactions. The infant further shows non-compliant behaviour and partially excludes the mother from the task / his/her game.
6. High. Infant’s anger is a predominant aspect of their interactions, but it is shown in more sporadic and generally subtler ways than in #7. Infant does not allow the mother to participate, but gives in after a few nudges.
7. Very High. Infant is repeatedly and overtly angry or resistant toward the parent. The degree of anger here seems so strong that the infant cannot disguise it in subtler ways for long, but it repeatedly appears in her/his interactions with him/her. The infant excludes the mother from the task / his/her game and does not want to solve the task / play together.
8. **Dyadic interaction scales**
9. Affective mutuality / felt security (DAM)

This scale assesses availability and mutuality of emotion between the infant and parent and how secure the infant feels with the parent. There is an emphasis on the infant having a sense that the parent has his/her own best interests in mind. There is also an emphasis on verbal and non-verbal communication, what the parent and infant communicate and how they do it. Open and free communication will be marked by emotion exchanged and a sense of personal involvement and engagement. The infant appears free to express positive or negative emotions or feelings. Availability of affect is also marked by the parent’s tone of voice communicating warmth and regard for the infant. At the low end, closed communication or lack of mutuality will be reflected in interaction that is stifled or non-reciprocal. At the low end there may be a veneer of intimacy or mutuality covering an impoverished experience; emotional experience of the parent may be quite different from experience of the infant. The rater must be alert to exchange of emotion and the subtle cues that reflect this. Essentially this scale is focused on behaviours which reflect on intimacy in the dyad. Dyads high on this scale almost always have a moment of shared emotion that is pleasurable. At the low end we see stifling of emotion, dampening behaviours which avoid or negate expression of emotion, or lots of conflict between the parent and the infant. The rater will need to distinguish between affect that is muted because of the parent’s focus on task (but which still regards infant’s feelings) and that which has as its purpose to stifle expression. Does parent respond to infant’s emotions and vice versa? Are there personal exchanges, eye contact? Does emotion and communication flow freely? Are positive emotions shared with one another?

1. Very High. There is a sense that experiences (both positive and negative) are shared, that the parent shows a response to the infant’s emotion and vice versa. Smiling back and forth takes place. Eye contact occurs when the infant or parent seeks it. There may also be physical proximity seeking behaviours, help seeking, that are responded to in a fashion that supports the mutuality observed in the dyad. There are almost no “dampening” behaviours by either partner, so that emotion and communication flows freely. There is at least one sustained bout of reciprocally communicated, positive emotion shared by the partners.
2. High. Very similar to #1 though a somewhat less active and overt exchange of emotions is noted. There may be a few ‘dampening’ behaviours when the infant shows negative affect (parent looks away or diverts attention) or when parent focuses heavily on instruction, but generally the infant feels understood. The dyad interacts in a relaxed fashion even if there is not a lot of eye contact, etc. There is an underlying warmth and appreciation between the two that is expressed even without lots of overt signs.
3. Moderately High. Brief periods of conflict or avoidance may be noted in an otherwise relaxed interaction, or parent and infant may have one or two interchanges in which emotional experience differs (e.g., upset infant, happy parent), but there is an attempt to reconcile experience.
4. Moderate. These dyads show a mixture of warmth and more restrictive or tense behaviours. There may be moments of tension and disengagement. Parent may seem a bit uneasy/flustered if the infant expresses frustration or anger and there may be an effort to “accentuate the positive” despite the infant’s needs to have feelings expressed. Dampening messages may be given, usually in a covert manner. Despite bouts of tension, however, there is a sense the dyad also likes each other, but that they are struggling a bit to figure it out.
5. Moderately Low. There are no bouts of sustained emotion shared between the two; instead, there is an increased emphasis on avoidance of emotion, negative emotion, and especially, non-mutual emotion. The parent may ignore or discourage the infant’s expression of emotion. The infant’s experience begins to take on an anxious quality, perhaps unsure that s/he can count on parent for assistance. The infant rarely initiates bids for security or parent affect. There are also moments of warmth but these are fleeting and occur under minimal stress.

2. Low. These dyads may seem cold or emotionless (like 7) but with some expressiveness and warmth at limited times or, they may be conflicted. Parents may be threatened by infant’s emotion and there are signs of disengagement or conflict when infant needs the parent. Parent may show signs of being annoyed or upset with the infant (angry look).

1. Very Low. There are three possibilities: 1) the dyad interaction seems staged apart from when there is a positive experience, e.g., smiling or laughter; 2) there is underlying conflict or ambivalence apparent (parent may make it clear he or she would rather be somewhere else); or 3) parent and infant have very little coordinated emotion and appear emotionally disconnected with each other. There may be underlying tension in the interaction. Parent may be threatened by any negative emotion. Dampening statements may not even be common since this dyad may essentially be disengaged around emotion. They may be highly engaged around the task / game or around performance but not emotion. There is very little attention to each other in terms of warmth or personal involvement. One may also see a parent giving derogatory glances at the infant, directly or indirectly communicating displeasure with the infant and/or his/her performance.
2. Reciprocity (DREC)

Are dialogues, bouts of interaction, and turn taking characterized by contingent responsiveness and engagement on the part of both parent and infant? Contingent responsiveness is indicated by appropriate, well-timed behavioural or verbal responses to comments, questions, or suggestions on the part of the parent and/or the infant. A “turn taking” (i.e. conversation-like) quality of interaction; behavioural flow.

1. No evidence of reciprocity. Parent and infant constantly interrupt one another.
2. One or two instances of reciprocity - either shared affect or eye contact.
3. A few/several instances of reciprocity (either shared affect or eye contact). The pair occasionally carries on reciprocal conversations, but these instances are never sustained.
4. Moderate levels of reciprocity; evidence of both shared affect and eye contact; some evidence of “conversation-like” interaction. Reciprocal interactions may be one-sided (i.e., mother makes suggestions, infant responds, or infant drives interaction, mother goes along) rather than true “turn-taking.”
5. Clear evidence of reciprocity; one or two episodes of intense shared positive affect coupled with eye contact that is sustained for several “turns” between mother and infant.
6. Substantial reciprocity involving numerous episodes of intense shared positive affect coupled with eye contact that is sustained for several “turns”; only one or two instances of non-reciprocity.
7. Highly integrated and reciprocal - constant shared positive affect and eye contact that never loses “turn taking” quality. There is a sense of back-and-forth interaction, communicated by looks, verbal expressions, or actions, and/or the cause-and-effect of behaviours or verbalizations is clear to the infant.

**Scales reliability**

Table S1 reports Cronbach’s alpha for the Beck Depression Inventory, 2^nd^ Edition (BDI-II, Beck et al., 1996) at each assessment stage. Table S2 reports Cronbach’s alpha for the scales Negative Affect, Distress, Falling Reactivity, Surgency and Orienting/Regulatory Capacity on the Infant Behavior Questionnaire – Revised, Very Short Form (IBQ-R VSF; Putnam et al., 2014) and Infant Behavior Questionnaire – Revised (IBQ-R; Gartstein & Rothbart, 2003) at each assessment stage. Please note that the low Cronbach’s alpha for the scales of Distress and Falling Reactivity at 2 weeks of age might be due to some of the items not being applicable at this young age (e.g. “After sleeping, how often did the baby play quietly in the crib?”; “When your baby was upset about something, how often did s/he soothe her/himself with other things (such as a stuffed animal, or blanket)?”).

**Table S1.** Cronbach’s alpha for maternal BDI-II scores at 2 weeks, 4 months, 6 months, and 9 months postpartum

|  | *2 Weeks* | *4 Months* | *6 Months* | *9 Months* |
| --- | --- | --- | --- | --- |
| *BDI-II* | .87 | .92 | .93 | .94 |

**Table S2.** Cronbach’s alpha for Negative Affect, Distress, Falling Reactivity, Surgency and Orienting / Regulatory Capacity scale scores on the Infant Behavior Questionnaire – Revised, Very Short Form (IBQ-R VSF; Putnam et al., 2014) and Infant Behavior Questionnaire – Revised (IBQ-R; Gartstein & Rothbart, 2003) at each assessment stage

|  | *Negative Affect* | *Distress* | *Falling Reactivity* | *Surgency* | *Orienting / Regulatory Capacity* |
| --- | --- | --- | --- | --- | --- |
| *2 weeks* | .878 | .585 | .626 | N/A | .796 |
| *4 months* | .791 | .664 | .845 | .913 | .620 |
| *6 months* | .815 | .696 | .812 | .696 | .726 |
| *9 months* | .813 | .611 | .825 | .778 | .697 |

*Note.* The items of the Surgency scale are not applicable at 2 weeks of age; scores are therefore not included here.

**Correlations between the interaction coding scales**

**Table S3.** Correlations between the interaction coding scales of maternal behaviour (Maternal Supportive Presence and Maternal Respect for Infant Autonomy), infant behaviour (Infant Autonomy and Infant Negativity), and dyadic behaviour (Dyadic Affective Mutuality and Dyadic Reciprocity)

|  | *MSP* | *MRIA* | *IA* | *IN* | *DAM* | *DREC* |
| --- | --- | --- | --- | --- | --- | --- |
| *MSP* | - |  |  |  |  |  |
| *MRIA* | **.518***** | - |  |  |  |  |
| *IA* | -.153 | -.230 | - |  |  |  |
| *IN* | -.133 | -.216 | -.027 | - |  |  |
| *DAM* | .328* | .080 | **.417***** | -.294* | - |  |
| *DREC* | .427** | .187 | **.454***** | -.232 | **.568***** | - |

*Note*. Significant correlations after Bonferroni correction are in bold. *p<.05, **p<.01, ***p<.001.

**Correlations between Interaction Behaviours and Maternal Depressive Symptoms**

**Table S4**. Correlations between the interaction behaviours derived from the interaction coding scales (Maternal Supportive Presence, Maternal Respect for Infant Autonomy, Infant Autonomy, Infant Negativity, Dyadic Affective Mutuality and Dyadic Reciprocity) and maternal depressive symptoms as assessed by the BDI-II (Beck et al., 1996).

|  | *MSP* | *MRIA* | *IA* | *IN* | *DAM* | *DREC* |
| --- | --- | --- | --- | --- | --- | --- |
| *BDI-II scores* | -.131 | -.258 | .199 | .219 | -.111 | .158 |

**Multiple Regression Models: Associations between Interaction Behaviours and Infant Temperament**

**Table S5.** Associations between Interaction Behaviours and Infant Temperament

| *Model Fit Measures for Maternal Supportive Presence (MSP)* | | | | | | | | | | | | | | | |
| --- | --- | --- | --- | --- | --- | --- | --- | --- | --- | --- | --- | --- | --- | --- | --- |
|  | | | | | | | | **Overall Model Test** | | | | | | | |
| **Model** | | **R** | | **R²** | | **Adjusted R²** | | **F** | | **df1** | | **df2** | | **p** | |
| 1 |  | 0.319 |  | 0.102 |  | 0.00818 |  | 1.09 |  | 5 |  | 48 |  | 0.379 |  |
|  | | | | | | | | | | | | | | | |

| *Multiple Regression Coefficients for Maternal Supportive Presence (MSP)* | | | | | | | | | |
| --- | --- | --- | --- | --- | --- | --- | --- | --- | --- |
| **Predictor** | | **Estimate** | | **SE** | | **t** | | **p** | |
| Intercept |  | 2.8213 |  | 1.652 |  | 1.708 |  | 0.094 |  |
| Negative Affect |  | -0.0247 |  | 0.209 |  | -0.118 |  | 0.906 |  |
| Surgency |  | -0.0967 |  | 0.235 |  | -0.411 |  | 0.683 |  |
| Orienting / Regulatory Capacity |  | 0.1468 |  | 0.240 |  | 0.612 |  | 0.544 |  |
| Distress |  | -0.0400 |  | 0.297 |  | -0.134 |  | 0.894 |  |
| Falling Reactivity |  | 0.2526 |  | 0.191 |  | 1.320 |  | 0.193 |  |
|  | | | | | | | | | |

| *Model Fit Measures for Maternal Respect of Infant Autonomy (MRIA)* | | | | | | | | | | | | | | | |
| --- | --- | --- | --- | --- | --- | --- | --- | --- | --- | --- | --- | --- | --- | --- | --- |
|  | | | | | | | | **Overall Model Test** | | | | | | | |
| **Model** | | **R** | | **R²** | | **Adjusted R²** | | **F** | | **df1** | | **df2** | | **p** | |
| 1 |  | 0.258 |  | 0.0665 |  | -0.0307 |  | 0.684 |  | 5 |  | 48 |  | 0.638 |  |
|  | | | | | | | | | | | | | | | |

| *Multiple Regression Coefficients for Maternal Respect of Infant Autonomy (MRIA)* | | | | | | | | | |
| --- | --- | --- | --- | --- | --- | --- | --- | --- | --- |
| **Predictor** | | **Estimate** | | **SE** | | **t** | | **p** | |
| Intercept |  | 3.5858 |  | 1.569 |  | 2.2861 |  | 0.027 |  |
| Negative Affect |  | 0.0167 |  | 0.198 |  | 0.0842 |  | 0.933 |  |
| Surgency |  | -0.1122 |  | 0.223 |  | -0.5023 |  | 0.618 |  |
| Orienting / Regulatory Capacity |  | -0.1117 |  | 0.228 |  | -0.4901 |  | 0.626 |  |
| Distress |  | 0.1293 |  | 0.282 |  | 0.4224 |  | 0.675 |  |
| Falling Reactivity |  | 0.3124 |  | 0.181 |  | 1.7202 |  | 0.092 |  |
|  | | | | | | | | | |

| *Model Fit Measures for Infant Agency / Autonomy (IA)* | | | | | | | | | | | | | | | |
| --- | --- | --- | --- | --- | --- | --- | --- | --- | --- | --- | --- | --- | --- | --- | --- |
|  | | | | | | | | **Overall Model Test** | | | | | | | |
| **Model** | | **R** | | **R²** | | **Adjusted R²** | | **F** | | **df1** | | **df2** | | **p** | |
| 1 |  | 0.475 |  | 0.225 |  | 0.144 |  | 2.79 |  | 5 |  | 48 |  | 0.027 |  |
|  | | | | | | | | | | | | | | | |

| *Multiple Regression Coefficients for Infant Agency / Autonomy (IA)* | | | | | | | | | |
| --- | --- | --- | --- | --- | --- | --- | --- | --- | --- |
| **Predictor** | | **Estimate** | | **SE** | | **t** | | **p** | |
| Intercept |  | -1.46780 |  | 1.797 |  | -0.81701 |  | 0.418 |  |
| Negative Affect |  | 0.16165 |  | 0.227 |  | 0.71135 |  | 0.480 |  |
| Surgency |  | 0.57561 |  | 0.256 |  | 2.25010 |  | 0.029 |  |
| Orienting / Regulatory Capacity |  | -0.00220 |  | 0.261 |  | -0.00842 |  | 0.993 |  |
| Distress |  | -0.15833 |  | 0.323 |  | -0.48952 |  | 0.627 |  |
| Falling Reactivity |  | 0.36078 |  | 0.208 |  | 1.73436 |  | 0.089 |  |

| *Model Fit Measures for Infant Negativity (IN)* | | | | | | | | | | | | | | | |
| --- | --- | --- | --- | --- | --- | --- | --- | --- | --- | --- | --- | --- | --- | --- | --- |
|  | | | | | | | | **Overall Model Test** | | | | | | | |
| **Model** | | **R** | | **R²** | | **Adjusted R²** | | **F** | | **df1** | | **df2** | | **p** | |
| 1 |  | 0.350 |  | 0.122 |  | 0.0309 |  | 1.34 |  | 5 |  | 48 |  | 0.265 |  |
|  | | | | | | | | | | | | | | | |

| *Multiple Regression Coefficients for Infant Negativity (IN)* | | | | | | | | | |
| --- | --- | --- | --- | --- | --- | --- | --- | --- | --- |
| **Predictor** | | **Estimate** | | **SE** | | **t** | | **p** | |
| Intercept |  | 2.4884 |  | 0.6974 |  | 3.568 |  | < .001 |  |
| Negative Affect |  | 0.0147 |  | 0.0882 |  | 0.166 |  | 0.869 |  |
| Surgency |  | 0.1660 |  | 0.0993 |  | 1.672 |  | 0.101 |  |
| Orienting / Regulatory Capacity |  | -0.1859 |  | 0.1013 |  | -1.835 |  | 0.073 |  |
| Distress |  | -0.1589 |  | 0.1256 |  | -1.266 |  | 0.212 |  |
| Falling Reactivity |  | -0.0775 |  | 0.0808 |  | -0.960 |  | 0.342 |  |
|  | | | | | | | | | |

| *Model Fit Measures for Dyadic Affective Mutuality (DAM)* | | | | | | | | | | | | | | | |
| --- | --- | --- | --- | --- | --- | --- | --- | --- | --- | --- | --- | --- | --- | --- | --- |
|  | | | | | | | | **Overall Model Test** | | | | | | | |
| **Model** | | **R** | | **R²** | | **Adjusted R²** | | **F** | | **df1** | | **df2** | | **p** | |
| 1 |  | 0.525 |  | 0.275 |  | 0.200 |  | 3.65 |  | 5 |  | 48 |  | **0.007** |  |
|  | | | | | | | | | | | | | | | |

| *Multiple Regression Coefficients for* *Dyadic Affective Mutuality (DAM)* | | | | | | | | | |
| --- | --- | --- | --- | --- | --- | --- | --- | --- | --- |
| **Predictor** | | **Estimate** | | **SE** | | **t** | | **p** | |
| Intercept |  | 1.9498 |  | 1.432 |  | 1.362 |  | 0.180 |  |
| Negative Affect |  | -0.2801 |  | 0.181 |  | -1.547 |  | 0.128 |  |
| Surgency |  | 0.1137 |  | 0.204 |  | 0.558 |  | 0.580 |  |
| Orienting / Regulatory Capacity |  | 0.0940 |  | 0.208 |  | 0.452 |  | 0.653 |  |
| Distress |  | 0.1792 |  | 0.258 |  | 0.695 |  | 0.490 |  |
| Falling Reactivity |  | 0.5067 |  | 0.166 |  | 3.057 |  | **0.004** |  |
|  | | | | | | | | | |

| *Model Fit Measures for Dyadic Reciprocity (DREC)* | | | | | | | | | | | | | | | |
| --- | --- | --- | --- | --- | --- | --- | --- | --- | --- | --- | --- | --- | --- | --- | --- |
|  | | | | | | | | **Overall Model Test** | | | | | | | |
| **Model** | | **R** | | **R²** | | **Adjusted R²** | | **F** | | **df1** | | **df2** | | **p** | |
| 1 |  | 0.594 |  | 0.352 |  | 0.285 |  | 5.22 |  | 5 |  | 48 |  | **< .001** |  |
|  | | | | | | | | | | | | | | | |

| *Multiple Regression Coefficients for Dyadic Reciprocity (DREC)* | | | | | | | | | |
| --- | --- | --- | --- | --- | --- | --- | --- | --- | --- |
| **Predictor** | | **Estimate** | | **SE** | | **t** | | **p** | |
| Intercept |  | -0.9950 |  | 1.196 |  | -0.832 |  | 0.409 |  |
| Negative Affect |  | 0.1721 |  | 0.151 |  | 1.138 |  | 0.261 |  |
| Surgency |  | 0.0527 |  | 0.170 |  | 0.309 |  | 0.758 |  |
| Orienting / Regulatory Capacity |  | 0.3617 |  | 0.174 |  | 2.083 |  | 0.043 |  |
| Distress |  | -0.1755 |  | 0.215 |  | -0.815 |  | 0.419 |  |
| Falling Reactivity |  | 0.2750 |  | 0.138 |  | 1.986 |  | 0.053 |  |
|  | | | | | | | | | |

*Note.* Maternal behaviour (Maternal Supportive Presence and Maternal Respect for Infant Autonomy), infant behaviour (Infant Autonomy and Infant Negativity), and dyadic behaviour (Dyadic Affective Mutuality and Dyadic Reciprocity) obtained from the interaction coding scales. Infant temperament scales obtained from the Infant Behavior Questionnaire–Revised – Very Short Form (IBQ-R VSF; Putnam et al., 2014) and from the Infant Behavior Questionnaire–Revised (IBQ-R; Gartstein & Rothbart, 2003). Significant associations are in bold.

**Additional analyses of the association between Dyadic Affective Mutuality (DAM) and Infant Falling Reactivity**

 An additional analysis was conducted to better understand at what time point(s) in the first months of life infant Falling Reactivity was significantly associated with DAM observed during the interaction at 9 months. To this aim, we ran correlations between DAM and Falling Reactivity at 2 weeks, 4, 6 and 9 months. All correlations were significant except the one involving Falling Reactivity at 2 weeks (see Table S6).

**Table S6.** Associations between infant Falling Reactivity at each time point and Dyadic Affective Mutuality observed at 9 months

|  | *Falling Reactivity* | | | |
| --- | --- | --- | --- | --- |
|  | *2 weeks* | *4 months* | *6 months* | *9 months* |
| *DAM at 9 months* | -.280 | **.381**** | **.546**** | **.326*** |

*Note.* Significant correlations are in bold. *p<.05, **p<.01

**Multiple Regression Models: Associations between Interaction Behaviours and Child Behavioural Outcomes**

**Table S7.** Associations between Interaction Behaviours and Child Behavioural Outcomes

| *Model Fit Measures for Maternal Supportive Presence (MSP)* | | | | | | | | | | | | | | | |
| --- | --- | --- | --- | --- | --- | --- | --- | --- | --- | --- | --- | --- | --- | --- | --- |
|  | | | | | | | | **Overall Model Test** | | | | | | | |
| **Model** | | **R** | | **R²** | | **Adjusted R²** | | **F** | | **df1** | | **df2** | | **p** | |
| 1 |  | 0.255 |  | 0.0648 |  | -0.0582 |  | 0.527 |  | 5 |  | 38 |  | 0.755 |  |
|  | | | | | | | | | | | | | | | |

| *Multiple Regression Coefficients for Maternal Supportive Presence (MSP)* | | | | | | | | | |
| --- | --- | --- | --- | --- | --- | --- | --- | --- | --- |
| **Predictor** | | **Estimate** | | **SE** | | **t** | | **p** | |
| Intercept |  | 4.3358 |  | 0.7338 |  | 5.908 |  | < .001 |  |
| Emotional Symptoms |  | -0.0153 |  | 0.1113 |  | -0.138 |  | 0.891 |  |
| Conduct Problems |  | -0.0711 |  | 0.1475 |  | -0.482 |  | 0.633 |  |
| Hyperactivity-Inattention |  | 0.0340 |  | 0.0928 |  | 0.366 |  | 0.716 |  |
| Peer Problems |  | -0.1130 |  | 0.0842 |  | -1.342 |  | 0.188 |  |
| Prosocial Behaviour |  | 0.0317 |  | 0.0778 |  | 0.407 |  | 0.686 |  |
|  | | | | | | | | | |

| *Model Fit Measures for Maternal Respect of Infant Autonomy (MRIA)* | | | | | | | | | | | | | | | |
| --- | --- | --- | --- | --- | --- | --- | --- | --- | --- | --- | --- | --- | --- | --- | --- |
|  | | | | | | | | **Overall Model Test** | | | | | | | |
| **Model** | | **R** | | **R²** | | **Adjusted R²** | | **F** | | **df1** | | **df2** | | **p** | |
| 1 |  | 0.515 |  | 0.265 |  | 0.168 |  | 2.74 |  | 5 |  | 38 |  | 0.033 |  |
|  | | | | | | | | | | | | | | | |

| *Multiple Regression Coefficients for* *Maternal Respect of Infant Autonomy (MRIA)* | | | | | | | | | |
| --- | --- | --- | --- | --- | --- | --- | --- | --- | --- |
| **Predictor** | | **Estimate** | | **SE** | | **t** | | **p** | |
| Intercept |  | 5.5519 |  | 0.5973 |  | 9.367 |  | < .001 |  |
| Emotional Symptoms |  | -0.0082 |  | 0.0906 |  | 0.188 |  | 0.929 |  |
| Conduct Problems |  | -0.1093 |  | 0.1201 |  | -1.012 |  | 0.368 |  |
| Hyperactivity-Inattention |  | 0.1140 |  | 0.0756 |  | 1.577 |  | 0.140 |  |
| Peer Problems |  | -0.2189 |  | 0.0685 |  | -3.291 |  | **0.003** |  |
| Prosocial Behaviour |  | -0.0645 |  | 0.0633 |  | -1.018 |  | 0.315 |  |
|  | | | | | | | | | |

| *Model Fit Measures for* *Infant Agency / Autonomy (IA)* | | | | | | | | | | | | | | | |
| --- | --- | --- | --- | --- | --- | --- | --- | --- | --- | --- | --- | --- | --- | --- | --- |
|  | | | | | | | | **Overall Model Test** | | | | | | | |
| **Model** | | **R** | | **R²** | | **Adjusted R²** | | **F** | | **df1** | | **df2** | | **p** | |
| 1 |  | 0.250 |  | 0.0627 |  | -0.0606 |  | 0.508 |  | 5 |  | 38 |  | 0.768 |  |
|  | | | | | | | | | | | | | | | |

| *Multiple Regression Coefficients for Infant Agency / Autonomy (IA)* | | | | | | | | | |
| --- | --- | --- | --- | --- | --- | --- | --- | --- | --- |
| **Predictor** | | **Estimate** | | **SE** | | **t** | | **p** | |
| Intercept |  | 2.3062 |  | 0.8493 |  | 2.715 |  | 0.010 |  |
| Emotional Symptoms |  | -0.0413 |  | 0.1289 |  | -0.321 |  | 0.750 |  |
| Conduct Problems |  | 0.0608 |  | 0.1707 |  | 0.356 |  | 0.724 |  |
| Hyperactivity-Inattention |  | -0.1396 |  | 0.1074 |  | -1.300 |  | 0.201 |  |
| Peer Problems |  | 0.0219 |  | 0.0975 |  | 0.224 |  | 0.824 |  |
| Prosocial Behaviour |  | 0.0850 |  | 0.0900 |  | 0.944 |  | 0.351 |  |
|  | | | | | | | | | |

| *Model Fit Measures for Infant Negativity (IN)* | | | | | | | | | | | | | | | |
| --- | --- | --- | --- | --- | --- | --- | --- | --- | --- | --- | --- | --- | --- | --- | --- |
|  | | | | | | | | **Overall Model Test** | | | | | | | |
| **Model** | | **R** | | **R²** | | **Adjusted R²** | | **F** | | **df1** | | **df2** | | **p** | |
| 1 |  | 0.296 |  | 0.0878 |  | -0.0322 |  | 0.732 |  | 5 |  | 38 |  | 0.604 |  |
|  | | | | | | | | | | | | | | | |

| *Multiple Regression Coefficients for Infant Negativity (IN)* | | | | | | | | | | | | |
| --- | --- | --- | --- | --- | --- | --- | --- | --- | --- | --- | --- | --- |
| **Predictor** | **Estimate** | | **SE** | | **t** | | | | **p** | | | |
| Intercept |  | 0.9713 |  | 0.3074 | |  | 3.160 |  | | 0.003 |  |  |
| Emotional Symptoms |  | -0.0627 |  | 0.0466 | |  | -1.344 |  | | 0.187 |  |  |
| Conduct Problems |  | 0.0540 |  | 0.0618 | |  | 0.875 |  | | 0.387 |  |  |
| Hyperactivity-Inattention |  | -0.0018 |  | 0.0389 | |  | -0.046 |  | | 0.963 |  |  |
| Peer Problems |  | 0.0284 |  | 0.0353 | |  | 0.806 |  | | 0.425 |  |  |
| Prosocial Behaviour |  | 0.0283 |  | 0.0326 | |  | 0.867 |  | | 0.391 |  |  |
|  | | | | | | | | | | | | |

| *Model Fit Measures for Dyadic Affective Mutuality (DAM)* | | | | | | | | | | | | | | | |
| --- | --- | --- | --- | --- | --- | --- | --- | --- | --- | --- | --- | --- | --- | --- | --- |
|  | | | | | | | | **Overall Model Test** | | | | | | | |
| **Model** | | **R** | | **R²** | | **Adjusted R²** | | **F** | | **df1** | | **df2** | | **p** | |
| 1 |  | 0.423 |  | 0.179 |  | 0.0707 |  | 1.65 |  | 5 |  | 38 |  | 0.169 |  |
|  | | | | | | | | | | | | | | | |

| *Multiple Regression Coefficients for Dyadic Affective Mutuality (DAM)* | | | | | | | | | |
| --- | --- | --- | --- | --- | --- | --- | --- | --- | --- |
| **Predictor** | | **Estimate** | | **SE** | | **t** | | **p** | |
| Intercept |  | 5.3544 |  | 0.6342 |  | 8.443 |  | < .001 |  |
| Emotional Symptoms |  | -0.0667 |  | 0.0962 |  | -0.693 |  | 0.492 |  |
| Conduct Problems |  | -0.0118 |  | 0.1275 |  | -0.092 |  | 0.927 |  |
| Hyperactivity-Inattention |  | -0.1859 |  | 0.0802 |  | -2.317 |  | 0.026 |  |
| Peer Problems |  | -0.0104 |  | 0.0728 |  | -0.143 |  | 0.887 |  |
| Prosocial Behaviour |  | 0.0489 |  | 0.0672 |  | 0.728 |  | 0.471 |  |
|  | | | | | | | | | |

| *Model Fit Measures for Dyadic Reciprocity (DREC)* | | | | | | | | | | | | | | | |
| --- | --- | --- | --- | --- | --- | --- | --- | --- | --- | --- | --- | --- | --- | --- | --- |
|  | | | | | | | | **Overall Model Test** | | | | | | | |
| **Model** | | **R** | | **R²** | | **Adjusted R²** | | **F** | | **df1** | | **df2** | | **p** | |
| 1 |  | 0.177 |  | 0.0312 |  | -0.0963 |  | 0.245 |  | 5 |  | 38 |  | 0.940 |  |
|  | | | | | | | | | | | | | | | |

| *Multiple Regression Coefficients for* *Dyadic Reciprocity (DREC)* | | | | | | | | | |
| --- | --- | --- | --- | --- | --- | --- | --- | --- | --- |
| **Predictor** | | **Estimate** | | **SE** | | **t** | | **p** | |
| Intercept |  | 2.3179 |  | 0.6354 |  | 3.6478 |  | < .001 |  |
| Emotional Symptoms |  | -0.0046 |  | 0.0964 |  | -0.0475 |  | 0.962 |  |
| Conduct Problems |  | 0.0133 |  | 0.1277 |  | 0.1038 |  | 0.918 |  |
| Hyperactivity-Inattention |  | -0.0283 |  | 0.0804 |  | -0.3517 |  | 0.727 |  |
| Peer Problems |  | -0.0502 |  | 0.0729 |  | -0.6881 |  | 0.496 |  |
| Prosocial Behaviour |  | 0.0459 |  | 0.0674 |  | 0.6810 |  | 0.500 |  |
|  | | | | | | | | | |

*Note.* Maternal behaviour (Maternal Supportive Presence and Maternal Respect for Infant Autonomy), infant behaviour (Infant Autonomy and Infant Negativity), and dyadic behaviour (Dyadic Affective Mutuality and Dyadic Reciprocity) obtained from the interaction coding scales. Child behavioural outcome obtained from the Strengths and Difficulties Questionnaire (SDQ, Goodman, 1997). Significant associations are in bold.

**RESULTS INCLUDING SOCIODEMOGRAPHIC VARIABLES**

Results from the analyses including sociodemographic variables (family annual income, maternal age, maternal years in education, and child gestational age) are presented below.

**Correlations between the interaction coding scales**

**Table S8.** Correlations between the interaction coding scales of maternal behaviour (Maternal Supportive Presence and Maternal Respect for Infant Autonomy), infant behaviour (Infant Autonomy and Infant Negativity), and dyadic behaviour (Dyadic Affective Mutuality and Dyadic Reciprocity), controlling for demographics of socio-economic status (family annual income, maternal age, maternal years in education, and child gestational age)

|  | *MSP* | *MRIA* | *IA* | *IN* | *DAM* | *DREC* |
| --- | --- | --- | --- | --- | --- | --- |
| *MSP* | - |  |  |  |  |  |
| *MRIA* | **.571***** | - |  |  |  |  |
| *IA* | -.117 | -.209 | - |  |  |  |
| *IN* | -.178 | -.217 | -.081 | - |  |  |
| *DAM* | .266 | .102 | **.470**** | -.278 | - |  |
| *DREC* | .450** | .232 | **.463**** | -.204 | **.488***** | - |

*Note*. Significant correlations after Bonferroni correction are in bold. *p<.05, **p<.01, ***p<.001.

**Correlations between Interaction Behaviours and Maternal Depressive Symptoms**

**Table S9**. Correlations between the interaction behaviours derived from the interaction coding scales (Maternal Supportive Presence, Maternal Respect for Infant Autonomy, Infant Autonomy, Infant Negativity, Dyadic Affective Mutuality and Dyadic Reciprocity) and maternal depressive symptoms as assessed by the BDI-II (Beck et al., 1996), controlling for demographics of socio-economic status (family annual income, maternal age, maternal years in education, and child gestational age)

|  | *MSP* | *MRIA* | *IA* | *IN* | *DAM* | *DREC* |
| --- | --- | --- | --- | --- | --- | --- |
| *BDI-II scores* | -.158 | -.297* | .018 | .335* | -.224 | .036 |

*Note*. Significant correlations after Bonferroni correction are in bold. *p<.05, **p<.01, ***p<.001.

**Multiple Regression Models: Associations between Interaction Behaviours and Infant Temperament**

**Table S10.** Associations between Interaction Behaviours derived from the interaction coding scales (Maternal Supportive Presence, Maternal Respect for Infant Autonomy, Infant Autonomy, Infant Negativity, Dyadic Affective Mutuality and Dyadic Reciprocity) and Infant Temperament obtained from the Infant Behavior Questionnaire–Revised – Very Short Form (IBQ-R VSF; Putnam et al., 2014) and from the Infant Behavior Questionnaire–Revised (IBQ-R; Gartstein & Rothbart, 2003), including demographics of socio-economic status (family annual income, maternal age, maternal years in education, and child gestational age)

| *Model Fit Measures for Maternal Supportive Presence (MSP)* | | | | | | | | | | | | | | | |
| --- | --- | --- | --- | --- | --- | --- | --- | --- | --- | --- | --- | --- | --- | --- | --- |
|  | | | | | | | | **Overall Model Test** | | | | | | | |
| **Model** | | **R** | | **R²** | | **Adjusted R²** | | **F** | | **df1** | | **df2** | | **p** | |
| 1 |  | 0.569 |  | 0.324 |  | 0.168 |  | 2.08 |  | 9 |  | 39 |  | 0.056 |  |
|  | | | | | | | | | | | | | | | |

| *Multiple Regression Coefficients for Maternal Supportive Presence (MSP)* | | | | | | | | | |
| --- | --- | --- | --- | --- | --- | --- | --- | --- | --- |
| **Predictor** | | **Estimate** | | **SE** | | **t** | | **p** | |
| Intercept |  | -0.1769 |  | 4.055 |  | -0.044 |  | 0.965 |  |
| Negative Affect |  | -0.0855 |  | 0.217 |  | -0.395 |  | 0.695 |  |
| Surgency |  | -0.0223 |  | 0.259 |  | -0.086 |  | 0.932 |  |
| Orienting / Regulatory Capacity |  | 0.1274 |  | 0.240 |  | 0.532 |  | 0.598 |  |
| Distress |  | 0.0023 |  | 0.282 |  | 0.008 |  | 0.993 |  |
| Falling Reactivity |  | 0.4136 |  | 0.192 |  | 2.157 |  | 0.037 |  |
| Maternal Age |  | -0.0088 |  | 0.029 |  | -0.307 |  | 0.761 |  |
| Family Income |  | 0.0000 |  | 0.000 |  | 2.471 |  | 0.018 |  |
| Maternal education |  | 0.0004 |  | 0.036 |  | 0.012 |  | 0.990 |  |
| Gestational Age |  | 0.0457 |  | 0.108 |  | 0.422 |  | 0.675 |  |
|  | | | | | | | | | |

| *Model Fit Measures for Maternal Respect of Infant Autonomy (MRIA)* | | | | | | | | | | | | | | | |
| --- | --- | --- | --- | --- | --- | --- | --- | --- | --- | --- | --- | --- | --- | --- | --- |
|  | | | | | | | | **Overall Model Test** | | | | | | | |
| **Model** | | **R** | | **R²** | | **Adjusted R²** | | **F** | | **df1** | | **df2** | | **p** | |
| 1 |  | 0.335 |  | 0.112 |  | -0.0927 |  | 0.548 |  | 9 |  | 39 |  | 0.831 |  |

| *Multiple Regression Coefficients for Maternal Respect of Infant Autonomy (MRIA)* | | | | | | | | | |
| --- | --- | --- | --- | --- | --- | --- | --- | --- | --- |
| **Predictor** | | **Estimate** | | **SE** | | **t** | | **p** | |
| Intercept |  | 4.0311 |  | 4.5153 |  | 0.8930 |  | 0.3770 |  |
| Negative Affect |  | 0.0975 |  | 0.2414 |  | 0.4040 |  | 0.6880 |  |
| Surgency |  | -0.0312 |  | 0.2885 |  | -0.1080 |  | 0.9140 |  |
| Orienting / Regulatory Capacity |  | -0.1413 |  | 0.2668 |  | -0.5300 |  | 0.5990 |  |
| Distress |  | 0.0834 |  | 0.3144 |  | 0.2650 |  | 0.7920 |  |
| Falling Reactivity |  | 0.2970 |  | 0.2135 |  | 1.3910 |  | 0.1720 |  |
| Maternal Age |  | 0.0281 |  | 0.0318 |  | 0.8820 |  | 0.3830 |  |
| Family Income |  | 0.0000 |  | 0.0000 |  | 1.2580 |  | 0.2160 |  |
| Maternal education |  | -0.0091 |  | 0.0399 |  | -0.2280 |  | 0.8210 |  |
| Gestational Age |  | -0.0451 |  | 0.1206 |  | -0.3740 |  | 0.7100 |  |
|  | | | | | | | | | |

| *Model Fit Measures for Infant Agency / Autonomy (IA)* | | | | | | | | | | | | | | | |
| --- | --- | --- | --- | --- | --- | --- | --- | --- | --- | --- | --- | --- | --- | --- | --- |
|  | | | | | | | | **Overall Model Test** | | | | | | | |
| **Model** | | **R** | | **R²** | | **Adjusted R²** | | **F** | | **df1** | | **df2** | | **p** | |
| 1 |  | 0.527 |  | 0.278 |  | 0.111 |  | 1.67 |  | 9 |  | 39 |  | 0.131 |  |
|  | | | | | | | | | | | | | | | |

| *Multiple Regression Coefficients for Infant Agency / Autonomy (IA)* | | | | | | | | | |
| --- | --- | --- | --- | --- | --- | --- | --- | --- | --- |
| **Predictor** | | **Estimate** | | **SE** | | **t** | | **p** | |
| Intercept |  | -6.6661 |  | 5.1491 |  | -1.2946 |  | 0.2030 |  |
| Negative Affect |  | -0.0048 |  | 0.2753 |  | -0.0173 |  | 0.9860 |  |
| Surgency |  | 0.5713 |  | 0.3289 |  | 1.7368 |  | 0.0900 |  |
| Orienting / Regulatory Capacity |  | -0.0119 |  | 0.3043 |  | -0.0391 |  | 0.9690 |  |
| Distress |  | -0.2233 |  | 0.3586 |  | -0.6227 |  | 0.5370 |  |
| Falling Reactivity |  | 0.3425 |  | 0.2435 |  | 1.4063 |  | 0.1680 |  |
| Maternal Age |  | -0.0235 |  | 0.0363 |  | -0.6476 |  | 0.5210 |  |
| Family Income |  | -4.13e−6 |  | 0.0000 |  | -0.7526 |  | 0.4560 |  |
| Maternal education |  | 0.0257 |  | 0.0455 |  | 0.5662 |  | 0.5750 |  |
| Gestational Age |  | 0.1701 |  | 0.1376 |  | 1.2364 |  | 0.2240 |  |

| *Model Fit Measures for Infant Negativity (IN)* | | | | | | | | | | | | | | | |
| --- | --- | --- | --- | --- | --- | --- | --- | --- | --- | --- | --- | --- | --- | --- | --- |
|  | | | | | | | | **Overall Model Test** | | | | | | | |
| **Model** | | **R** | | **R²** | | **Adjusted R²** | | **F** | | **df1** | | **df2** | | **p** | |
| 1 |  | 0.4 |  | 0.16 |  | -0.0335 |  | 0.827 |  | 9 |  | 39 |  | 0.595 |  |
|  | | | | | | | | | | | | | | | |

| *Multiple Regression Coefficients for Infant Negativity (IN)* | | | | | | | | | |
| --- | --- | --- | --- | --- | --- | --- | --- | --- | --- |
| **Predictor** | | **Estimate** | | **SE** | | **t** | | **p** | |
| Intercept |  | 1.6447 |  | 1.9345 |  | 0.8502 |  | 0.4000 |  |
| Negative Affect |  | -0.0374 |  | 0.1034 |  | -0.3616 |  | 0.7200 |  |
| Surgency |  | -0.0040 |  | 0.1236 |  | -0.0322 |  | 0.9740 |  |
| Orienting / Regulatory Capacity |  | -0.0903 |  | 0.1143 |  | -0.7901 |  | 0.4340 |  |
| Distress |  | -0.1101 |  | 0.1347 |  | -0.8169 |  | 0.4190 |  |
| Falling Reactivity |  | -0.1321 |  | 0.0915 |  | -1.4436 |  | 0.1570 |  |
| Maternal Age |  | -0.0090 |  | 0.0136 |  | -0.6628 |  | 0.5110 |  |
| Family Income |  | -2.69e−6 |  | 0.0000 |  | -1.3050 |  | 0.2000 |  |
| Maternal education |  | 0.0023 |  | 0.0171 |  | 0.1337 |  | 0.8940 |  |
| Gestational Age |  | 0.0423 |  | 0.0517 |  | 0.8185 |  | 0.4180 |  |
|  | | | | | | | | | |

| *Model Fit Measures for Dyadic Affective Mutuality (DAM)* | | | | | | | | | | | | | | | |
| --- | --- | --- | --- | --- | --- | --- | --- | --- | --- | --- | --- | --- | --- | --- | --- |
|  | | | | | | | | **Overall Model Test** | | | | | | | |
| **Model** | | **R** | | **R²** | | **Adjusted R²** | | **F** | | **df1** | | **df2** | | **p** | |
| 1 |  | 0.704 |  | 0.496 |  | 0.38 |  | 4.27 |  | 9 |  | 39 |  | **<0.001** |  |
|  | | | | | | | | | | | | | | | |

| *Multiple Regression Coefficients for Dyadic Affective Mutuality (DAM)* | | | | | | | | | |
| --- | --- | --- | --- | --- | --- | --- | --- | --- | --- |
| **Predictor** | | **Estimate** | | **SE** | | **t** | | **p** | |
| Intercept |  | -2.267 |  | 3.511 |  | -0.646 |  | 0.522 |  |
| Negative Affect |  | -0.395 |  | 0.188 |  | -2.102 |  | 0.042 |  |
| Surgency |  | 0.393 |  | 0.224 |  | 1.751 |  | 0.088 |  |
| Orienting / Regulatory Capacity |  | -0.078 |  | 0.208 |  | -0.376 |  | 0.709 |  |
| Distress |  | 0.179 |  | 0.245 |  | 0.730 |  | 0.470 |  |
| Falling Reactivity |  | 0.677 |  | 0.166 |  | 4.076 |  | **< .001** |  |
| Maternal Age |  | -0.004 |  | 0.025 |  | -0.168 |  | 0.867 |  |
| Family Income |  | 0.000 |  | 0.000 |  | 0.713 |  | 0.480 |  |
| Maternal education |  | 0.044 |  | 0.031 |  | 1.430 |  | 0.161 |  |
| Gestational Age |  | 0.073 |  | 0.094 |  | 0.782 |  | 0.439 |  |
|  | | | | | | | | | |

| *Model Fit Measures for Dyadic Reciprocity (DREC)* | | | | | | | | | | | | | | | |
| --- | --- | --- | --- | --- | --- | --- | --- | --- | --- | --- | --- | --- | --- | --- | --- |
|  | | | | | | | | **Overall Model Test** | | | | | | | |
| **Model** | | **R** | | **R²** | | **Adjusted R²** | | **F** | | **df1** | | **df2** | | **p** | |
| 1 |  | 0.718 |  | 0.516 |  | 0.404 |  | 4.62 |  | 9 |  | 39 |  | **< .001** |  |
|  | | | | | | | | | | | | | | | |

| *Multiple Regression Coefficients for Dyadic Reciprocity (DREC)* | | | | | | | | | |
| --- | --- | --- | --- | --- | --- | --- | --- | --- | --- |
| **Predictor** | | **Estimate** | | **SE** | | **t** | | **p** | |
| Intercept |  | -5.4048 |  | 3.061 |  | -1.766 |  | 0.085 |  |
| Negative Affect |  | 0.1389 |  | 0.1636 |  | 0.849 |  | 0.401 |  |
| Surgency |  | 0.2894 |  | 0.1956 |  | 1.48 |  | 0.147 |  |
| Orienting / Regulatory Capacity |  | 0.1852 |  | 0.1809 |  | 1.024 |  | 0.312 |  |
| Distress |  | -0.1684 |  | 0.2132 |  | -0.79 |  | 0.434 |  |
| Falling Reactivity |  | 0.3878 |  | 0.1448 |  | 2.679 |  | 0.011 |  |
| Maternal Age |  | 0.015 |  | 0.0216 |  | 0.693 |  | 0.492 |  |
| Family Income |  | 2.26E-06 |  | 3.26E-06 |  | 0.692 |  | 0.493 |  |
| Maternal education |  | 0.0415 |  | 0.027 |  | 1.536 |  | 0.133 |  |
| Gestational Age |  | 0.0672 |  | 0.0818 |  | 0.821 |  | 0.417 |  |
|  | | | | | | | | | |

*Note.* Significant associations are in bold.

**Multiple Regression Models: Associations between Interaction Behaviours and Child Behavioural Outcomes**

**Table S11.** Associations between Interaction Behaviours (Maternal Supportive Presence, Maternal Respect for Infant Autonomy, Infant Autonomy, Infant Negativity, Dyadic Affective Mutuality and Dyadic Reciprocity) and Child Behavioural Outcomes obtained from the Strengths and Difficulties Questionnaire (SDQ, Goodman, 1997), including demographics of socio-economic status (family annual income, maternal age, maternal years in education, and child gestational age)

| *Model Fit Measures for Maternal Supportive Presence (MSP)* | | | | | | | | | | | | | | | |
| --- | --- | --- | --- | --- | --- | --- | --- | --- | --- | --- | --- | --- | --- | --- | --- |
|  | | | | | | | | **Overall Model Test** | | | | | | | |
| **Model** | | **R** | | **R²** | | **Adjusted R²** | | **F** | | **df1** | | **df2** | | **p** | |
| 1 |  | 0.503 |  | 0.253 |  | 0.0361 |  | 1.17 |  | 9 |  | 31 |  | 0.350 |  |
|  | | | | | | | | | | | | | | | |

| *Multiple Regression Coefficients for Maternal Supportive Presence (MSP)* | | | | | | | | | |
| --- | --- | --- | --- | --- | --- | --- | --- | --- | --- |
| **Predictor** | | **Estimate** | | **SE** | | **t** | | **p** | |
| Intercept |  | 6.0520 |  | 5.3823 |  | 1.1240 |  | 0.2690 |  |
| Emotional Symptoms |  | -0.0597 |  | 0.1137 |  | -0.5250 |  | 0.6030 |  |
| Conduct Problems |  | -0.0601 |  | 0.1503 |  | -0.3990 |  | 0.6920 |  |
| Hyperactivity-Inattention |  | 0.0635 |  | 0.0934 |  | 0.6800 |  | 0.5010 |  |
| Peer Problems |  | -0.1280 |  | 0.0893 |  | -1.4340 |  | 0.1620 |  |
| Prosocial Behaviour |  | 0.0709 |  | 0.0792 |  | 0.8960 |  | 0.3770 |  |
| Maternal Age |  | 0.0113 |  | 0.0344 |  | 0.3300 |  | 0.7440 |  |
| Family Income |  | 0.0000 |  | 0.0000 |  | 1.6860 |  | 0.1020 |  |
| Maternal education |  | 0.0077 |  | 0.0413 |  | 0.1870 |  | 0.8530 |  |
| Gestational Age |  | -0.0741 |  | 0.1271 |  | -0.5830 |  | 0.5640 |  |
|  | | | | | | | | | |

| *Model Fit Measures for Maternal Respect of Infant Autonomy (MRIA)* | | | | | | | | | | | | | | | |
| --- | --- | --- | --- | --- | --- | --- | --- | --- | --- | --- | --- | --- | --- | --- | --- |
|  | | | | | | | | **Overall Model Test** | | | | | | | |
| **Model** | | **R** | | **R²** | | **Adjusted R²** | | **F** | | **df1** | | **df2** | | **p** | |
| 1 |  | 0.555 |  | 0.308 |  | 0.107 |  | 1.53 |  | 9 |  | 31 |  | 0.181 |  |
|  | | | | | | | | | | | | | | | |

| *Multiple Regression Coefficients for* *Maternal Respect of Infant Autonomy (MRIA)* | | | | | | | | | |
| --- | --- | --- | --- | --- | --- | --- | --- | --- | --- |
| **Predictor** | | **Estimate** | | **SE** | | **t** | | **p** | |
| Intercept |  | 11.7392 |  | 4.9855 |  | 2.3550 |  | 0.025 |  |
| Emotional Symptoms |  | 0.0275 |  | 0.1054 |  | 0.2610 |  | 0.796 |  |
| Conduct Problems |  | -0.0715 |  | 0.1392 |  | -0.5140 |  | 0.611 |  |
| Hyperactivity-Inattention |  | 0.1033 |  | 0.0865 |  | 1.1940 |  | 0.242 |  |
| Peer Problems |  | -0.2523 |  | 0.0827 |  | -3.0520 |  | **0.005** |  |
| Prosocial Behaviour |  | -0.0304 |  | 0.0733 |  | -0.4150 |  | 0.681 |  |
| Maternal Age |  | 0.0107 |  | 0.0319 |  | 0.3350 |  | 0.740 |  |
| Family Income |  | 0.0000 |  | 0.0000 |  | 0.4010 |  | 0.691 |  |
| Maternal education |  | -0.0276 |  | 0.0382 |  | -0.7210 |  | 0.476 |  |
| Gestational Age |  | -0.1603 |  | 0.1178 |  | -1.3610 |  | 0.183 |  |
|  | | | | | | | | | |

| *Model Fit Measures for* *Infant Agency / Autonomy (IA)* | | | | | | | | | | | | | | | |
| --- | --- | --- | --- | --- | --- | --- | --- | --- | --- | --- | --- | --- | --- | --- | --- |
|  | | | | | | | | **Overall Model Test** | | | | | | | |
| **Model** | | **R** | | **R²** | | **Adjusted R²** | | **F** | | **df1** | | **df2** | | **p** | |
| 1 |  | 0.543 |  | 0.295 |  | -0.0906 |  | 1.44 |  | 9 |  | 31 |  | 0.213 |  |
|  | | | | | | | | | | | | | | | |

| *Multiple Regression Coefficients for Infant Agency / Autonomy (IA)* | | | | | | | | | |
| --- | --- | --- | --- | --- | --- | --- | --- | --- | --- |
| **Predictor** | | **Estimate** | | **SE** | | **t** | | **p** | |
| Intercept |  | -8.51004 |  | 6.3795 |  | -1.334 |  | 0.192 |  |
| Emotional Symptoms |  | -0.05696 |  | 0.1348 |  | -0.4225 |  | 0.676 |  |
| Conduct Problems |  | -0.076 |  | 0.1782 |  | -0.4266 |  | 0.673 |  |
| Hyperactivity-Inattention |  | -0.16524 |  | 0.1107 |  | -1.4927 |  | 0.146 |  |
| Peer Problems |  | 0.08615 |  | 0.1058 |  | 0.8143 |  | 0.422 |  |
| Prosocial Behaviour |  | -0.0029 |  | 0.0939 |  | -0.0309 |  | 0.976 |  |
| Maternal Age |  | -0.03766 |  | 0.0408 |  | -0.9231 |  | 0.363 |  |
| Family Income |  | -9.55e−6 |  | 6.03E-06 |  | -1.583 |  | 0.124 |  |
| Maternal education |  | 0.04443 |  | 0.0489 |  | 0.908 |  | 0.371 |  |
| Gestational Age |  | 0.31627 |  | 0.1507 |  | 2.099 |  | 0.044 |  |
|  | | | | | | | | | |

| *Model Fit Measures for Infant Negativity (IN)* | | | | | | | | | | | | | | | |
| --- | --- | --- | --- | --- | --- | --- | --- | --- | --- | --- | --- | --- | --- | --- | --- |
|  | | | | | | | | **Overall Model Test** | | | | | | | |
| **Model** | | **R** | | **R²** | | **Adjusted R²** | | **F** | | **df1** | | **df2** | | **p** | |
| 1 |  | 0.38 |  | 0.145 |  | -0.104 |  | 0.583 |  | 9 |  | 31 |  | 0.801 |  |
|  | | | | | | | | | | | | | | | |

| *Multiple Regression Coefficients for Infant Negativity (IN)* | | | | | | | | | | | | |
| --- | --- | --- | --- | --- | --- | --- | --- | --- | --- | --- | --- | --- |
| **Predictor** | **Estimate** | | **SE** | | **t** | | | | **p** | | | |
| Intercept |  | 0.8482 |  | 2.5496 | |  | 0.3330 |  | | 0.7420 |  |  |
| Emotional Symptoms |  | -0.0560 |  | 0.0539 | |  | -1.0400 |  | | 0.3070 |  |  |
| Conduct Problems |  | 0.0181 |  | 0.0712 | |  | 0.2530 |  | | 0.8020 |  |  |
| Hyperactivity-Inattention |  | -0.0067 |  | 0.0442 | |  | -0.1500 |  | | 0.8810 |  |  |
| Peer Problems |  | 0.0268 |  | 0.0423 | |  | 0.6330 |  | | 0.5310 |  |  |
| Prosocial Behaviour |  | 0.0137 |  | 0.0375 | |  | 0.3650 |  | | 0.7180 |  |  |
| Maternal Age |  | -0.0201 |  | 0.0163 | |  | -1.2300 |  | | 0.2280 |  |  |
| Family Income |  | -7.59e−7 |  | 0.0000 | |  | -0.3150 |  | | 0.7550 |  |  |
| Maternal education |  | -0.0048 |  | 0.0196 | |  | -0.2470 |  | | 0.8060 |  |  |
| Gestational Age |  | 0.0275 |  | 0.0602 | |  | 0.4570 |  | | 0.6510 |  |  |
|  | | | | | | | | | | | | |

| *Model Fit Measures for Dyadic Affective Mutuality (DAM)* | | | | | | | | | | | | | | | |
| --- | --- | --- | --- | --- | --- | --- | --- | --- | --- | --- | --- | --- | --- | --- | --- |
|  | | | | | | | | **Overall Model Test** | | | | | | | |
| **Model** | | **R** | | **R²** | | **Adjusted R²** | | **F** | | **df1** | | **df2** | | **p** | |
| 1 |  | 0.53 |  | 0.281 |  | 0.0727 |  | 1.35 |  | 9 |  | 31 |  | 0.253 |  |
|  | | | | | | | | | | | | | | | |

| *Multiple Regression Coefficients for Dyadic Affective Mutuality (DAM)* | | | | | | | | | |
| --- | --- | --- | --- | --- | --- | --- | --- | --- | --- |
| **Predictor** | | **Estimate** | | **SE** | | **t** | | **p** | |
| Intercept |  | 2.2457 |  | 5.0840 |  | 0.4417 |  | 0.662 |  |
| Emotional Symptoms |  | -0.1310 |  | 0.1074 |  | -1.2189 |  | 0.232 |  |
| Conduct Problems |  | -0.0154 |  | 0.1420 |  | -0.1087 |  | 0.914 |  |
| Hyperactivity-Inattention |  | -0.1793 |  | 0.0882 |  | -2.0322 |  | 0.051 |  |
| Peer Problems |  | 0.0216 |  | 0.0843 |  | 0.2557 |  | 0.800 |  |
| Prosocial Behaviour |  | 0.0478 |  | 0.0748 |  | 0.6384 |  | 0.528 |  |
| Maternal Age |  | 0.0020 |  | 0.0325 |  | 0.0602 |  | 0.952 |  |
| Family Income |  | 0.0000 |  | 0.0000 |  | 0.1579 |  | 0.876 |  |
| Maternal education |  | 0.0700 |  | 0.0390 |  | 1.7960 |  | 0.082 |  |
| Gestational Age |  | 0.0450 |  | 0.1201 |  | 0.3750 |  | 0.710 |  |
|  | | | | | | | | | |

| *Model Fit Measures for Dyadic Reciprocity (DREC)* | | | | | | | | | | | | | | | |
| --- | --- | --- | --- | --- | --- | --- | --- | --- | --- | --- | --- | --- | --- | --- | --- |
|  | | | | | | | | **Overall Model Test** | | | | | | | |
| **Model** | | **R** | | **R²** | | **Adjusted R²** | | **F** | | **df1** | | **df2** | | **p** | |
| 1 |  | 0.477 |  | 0.227 |  | -0.00294 |  | 1.01 |  | 9 |  | 31 |  | 0.451 |  |
|  | | | | | | | | | | | | | | | |

| *Multiple Regression Coefficients for* *Dyadic Reciprocity (DREC)* | | | | | | | | | |
| --- | --- | --- | --- | --- | --- | --- | --- | --- | --- |
| **Predictor** | | **Estimate** | | **SE** | | **t** | | **p** | |
| Intercept |  | -5.8351 |  | 4.9084 |  | -1.1888 |  | 0.244 |  |
| Emotional Symptoms |  | -0.0889 |  | 0.1037 |  | -0.8572 |  | 0.398 |  |
| Conduct Problems |  | 0.0018 |  | 0.1371 |  | 0.0129 |  | 0.990 |  |
| Hyperactivity-Inattention |  | 0.0075 |  | 0.0852 |  | 0.0874 |  | 0.931 |  |
| Peer Problems |  | -0.0098 |  | 0.0814 |  | -0.1208 |  | 0.905 |  |
| Prosocial Behaviour |  | 0.0570 |  | 0.0722 |  | 0.7898 |  | 0.436 |  |
| Maternal Age |  | 0.0341 |  | 0.0314 |  | 1.0849 |  | 0.286 |  |
| Family Income |  | -1.77e−6 |  | 0.0000 |  | -0.3818 |  | 0.705 |  |
| Maternal education |  | 0.0694 |  | 0.0377 |  | 1.8435 |  | 0.075 |  |
| Gestational Age |  | 0.1453 |  | 0.1159 |  | 1.2534 |  | 0.219 |  |
|  | | | | | | | | | |

*Note.* Significant associations are in bold.

**REFERENCES**

Beck, A.T., Steer, R.A., & Brown, G.K. (1996). *Manual for the Beck Depression Inventory-II*. San Antonio, TX: Psychological Corporation.

Gartstein, M. A., & Rothbart, M. K. (2003). Studying infant temperament via the Revised Infant Behavior Questionnaire. *Infant Behavior and Development, 26(1)*, 64-86.

Goodman, R. (1997). The Strengths and Difficulties Questionnaire: a research note. *Journal of Child Psychology and Psychiatry, 38*(5), 581-586.

Putnam, S. P., Helbig, A. L., Gartstein, M. A., Rothbart, M. K., & Leerkes, E. (2014). Development and Assessment of Short and very Short Forms of the Infant Behavior Questionnaire-Revised. *Journal of Personality Assessment*, 1-14.
