# Supplementary material for: Mother-infant interaction characteristics associate with infant falling reactivity and child peer problems at pre-school age
Source: PLoS One. 2024 Jun 4;19(6):e0302661. doi: 10.1371/journal.pone.0302661 (PMC11149888; doi:10.1371/journal.pone.0302661)
Supplement: Attachment — Submitted filename: Comments.docx [file pone.0302661.s002.docx]

**Reviewer’s comments:**

Thank you for the opportunity to review the manuscript, “Mother-infant interaction characteristics associate with infant falling reactivity and child peer problems at pre-school age”. PONE-D-23-29119

The topic of the manuscript is interesting, the quality of the early interaction between an infant and his or her mother is an important factor that influences the child’s development, survival and growth.  This type of research has been discussed repeatedly in previous literature; however, it is still needed to direct the plans for intervention procedures to alter mother–infant interaction with the ultimate goal of achieving positive developmental outcomes during the first years of life.

**The introduction**

The introduction must be revised as there are some outdated references. It is prolonged and full of details of previous studies. It included description of the tools which have been used in the study. These details must be transferred to the methodology section.

These are examples of recent references that can help in revising the introduction

- Rocha, N. A. C. F., Dos Santos Silva, F. P., Dos Santos, M. M., & Dusing, S. C. (2020). Impact of mother-infant interaction on development during the first year of life: A systematic review. *Journal of child health care: for professionals working with children in the hospital and community*, *24*(3), 365–385. <https://doi.org/10.1177/1367493519864742>
- Chung, FF., Wan, GH., Kuo, SC. *et al.* Mother–infant interaction quality and sense of parenting competence at six months postpartum for first-time mothers in Taiwan: a multiple time series design. *BMC Pregnancy Childbirth* **18**, 365 (2018). <https://doi.org/10.1186/s12884-018-1979-7>
- Richter M, Fehringer K, Smith J, Pineda R. Parent-infant interaction in the NICU: Challenges in measurement. Early Hum Dev. 2022 Jul;170:105609. doi: 10.1016/j.earlhumdev.2022.105609. Epub 2022 Jun 14. PMID: 35752043; PMCID: PMC10072234.

**Methods:**

This section comprises a lot of limitations

**Participants:**

The authors did not mention the basis for calculation of sample size.

Although authors described some features of the participants, they did not mention the exact inclusion and exclusion criteria of the sample. Neither the socioeconomic level nor the marital status of the participants mothers was elaborated; in spite of the known impact of these variables on the mother -infant interaction.

**Study design:**

The longitudinal study design allowed the authors to investigate the associations between mother-infant interaction characteristics at 9 months of age with maternal depression and infant temperament along the first year postpartum, and with child behavior at 3 years of age. It is important to consider the authors’ efforts to keep the sample intact over this time.

The authors mentioned that there were five points of assessment consisting of one pre- and four post-natal assessment points. However, they excluded the prenatal assessment point. I think it is important to include this assessment to avoid loss of the biological factors’ effect on the mother-infant interaction.

The authors must specify the time of the four post-natal assessment points in the section of methods. These points appeared for the first time in Table S1 in supporting information file.

The major limitation in methods was the single point assessment of the main outcome variable (mother-infant interaction). It was assessed once at the age of 9 months. This main variable must be evaluated as the average of several assessments at several points of time as the maternal depression and infant temperament. In addition, it was unsuitable to depend on the undergraduate students in evaluation of the mother-infant interaction. Instead, expert developmental and behavioral pediatricians had to do this evaluation.

The Strengths and Difficulties Questionnaire (SDQ) should not be used for this young age, many available questionnaires could be used to investigate the child’s behaviors, emotions and relationships at the age of 36 months. (e.g. Child Behavior Checklist (CBCL), Ages & Stages Questionnaire: Social-Emotional (ASQ: SE)

Confounding variables were not considered in this study, which might affect mother-infant relationship as health status of the infant or the mother, socioeconomic variables, social support, work status, and marital status.

**Results:**

Were well presented.

Table S6 in the supporting file: It described the associations between infant Falling Reactivity at each time point and Dyadic Affective Mutuality observed at 9 months. These associations were reported to be positive. Thus, authors have to revise for the negative sign in each correlation.

**Discussion:**

The finding of a lack of association between interaction behaviors and maternal depressive symptoms was not acceptable. This may be due to the dependence on single evaluation of interaction behavior as mentioned before; or because all participants had minimal depression symptoms that could not impact the interaction behavior. The other two findings are acceptable.
