# Supplementary material for: Mother-infant interaction characteristics associate with infant falling reactivity and child peer problems at pre-school age
Source: PLoS One. 2024 Jun 4;19(6):e0302661. doi: 10.1371/journal.pone.0302661 (PMC11149888; doi:10.1371/journal.pone.0302661)
Supplement: Attachment — Submitted filename: Response to Reviewers.docx [file pone.0302661.s003.docx]

PONE-D-23-29119

Mother-infant interaction characteristics associate with infant falling reactivity and child peer problems at pre-school age

PLOS ONE

Reviewers' comments:

Reviewer #1: Thank you for the opportunity to review the manuscript, “Mother-infant interaction characteristics associate with infant falling reactivity and child peer problems at pre-school age”. PONE-D-23-29119
The topic of the manuscript is interesting, the quality of the early interaction between an infant and his or her mother is an important factor that influences the child’s development, survival and growth. This type of research has been discussed repeatedly in previous literature; however, it is still needed to direct the plans for intervention procedures to alter mother–infant interaction with the ultimate goal of achieving positive developmental outcomes during the first years of life.

*Response: We would like to thank this reviewer for acknowledging the importance of our work.*

The introduction
1. The introduction must be revised as there are some outdated references. It is prolonged and full of details of previous studies. It included description of the tools which have been used in the study. These details must be transferred to the methodology section.
These are examples of recent references that can help in revising the introduction
• Rocha, N. A. C. F., Dos Santos Silva, F. P., Dos Santos, M. M., & Dusing, S. C. (2020). Impact of mother-infant interaction on development during the first year of life: A systematic review. Journal of child health care: for professionals working with children in the hospital and community, 24(3), 365–385. [https://doi.org/10.1177/1367493519864742](https://linkprotect.cudasvc.com/url?a=https%3a%2f%2fdoi.org%2f10.1177%2f1367493519864742&c=E,1,hO1cCDz6KzFW0v1ZIv9p2U4-kmgkNOKO4ALkRd7e6N2ZbId3-P1V0pLzoVUiLq36-R9Zgnw-rdrRcrGIemlyGt9Clr-OyLFXjGT692RlDsv8bgAMkhVu&typo=1&ancr_add=1)
• Chung, FF., Wan, GH., Kuo, SC. et al. Mother–infant interaction quality and sense of parenting competence at six months postpartum for first-time mothers in Taiwan: a multiple time series design. BMC Pregnancy Childbirth 18, 365 (2018). [https://doi.org/10.1186/s12884-018-1979-7](https://linkprotect.cudasvc.com/url?a=https%3a%2f%2fdoi.org%2f10.1186%2fs12884-018-1979-7&c=E,1,JeHXWEyw5ORoWH6e3ZuSZOKRqeLnpqu9xdNJ_AaJxA8MJSubVfkgcJuciiZgBfghdv4fm8cSSBd9RCY4OVahJccOMmORGW6rP2Na2XIj1rE8jyaqNQOdwQ,,&typo=1&ancr_add=1)
• Richter M, Fehringer K, Smith J, Pineda R. Parent-infant interaction in the NICU: Challenges in measurement. Early Hum Dev. 2022 Jul;170:105609. doi: 10.1016/j.earlhumdev.2022.105609. Epub 2022 Jun 14. PMID: 35752043; PMCID: PMC10072234.

*Response: We are grateful to this reviewer for suggesting additional references and improvements to the Introduction section. We added the reference to the review by Rocha et al. (2020) which we found very relevant. However, we felt that Chung et al. (2018) and Richter et al.’s (2022) studies are too specific to special populations and therefore not directly relevant for our study – i.e., first-time mothers and high-risk dyads in NICU. As further recommended by this reviewer, the Introduction section has now been shortened in several places (pp.4-6) and details pertaining to methods originally included at the end of the Introduction section have been moved and integrated in the Methods section (pp.13-14).*

Methods:
2. This section comprises a lot of limitations
Participants:
The authors did not mention the basis for calculation of sample size.

*Response: Thank you for this important comment. When we started this longitudinal study, no formal sample size calculation was used due to the lack of previous longitudinal studies with similar measures to base the calculation on. However, in cross­-sectional studies more generally, all the effects we were studying in terms of infants' attention and social abilities have been robustly found and replicated in samples much smaller (typically 10­-20 infants) than the one we originally proposed (approx. 60). Since no previous study had investigated performance on the tasks longitudinally, we felt that a larger sample was needed to accommodate for participant drop­out and the yet unknown magnitude of longitudinal stability during the first year of life.*

*The final sample size of our study was limited to some extent by the labour-intensive nature of carrying out longitudinal infant work (in terms of continuous participant contact, preparation for testing, testing with plenty of breaks to capture each infant's most alert and attentive state, and manual data analysis and coding of electrophysiological and behavioural data).*

*We have added this sentence on p.8-9. “No formal sample size calculation was used due to the lack of previous longitudinal studies with similar measures to base the calculation on. There were also financial and time constraints. However, a sample size of 54 infants is larger than most infant studies involving extensive behavioural testing.”*

3. Although authors described some features of the participants, they did not mention the exact inclusion and exclusion criteria of the sample. Neither the socioeconomic level nor the marital status of the participants mothers was elaborated; in spite of the known impact of these variables on the mother -infant interaction.

*Response: We are sorry for this omission. At time of recruitment of our longitudinal study, the inclusion criteria were that the potential participant was female, pregnant, and had not yet entered the 37th week of pregnancy. There were no exclusion criteria. We have added this information to the Participants section on p.7. In the same section, we also explain that 2 infants were excluded from the recruited sample due to complications at birth. This was the sole exclusion criterion at that stage.
The reviewer is right that sociodemographic variables may play a critical role in mother-infant interactions, and these are summarised in the methods section on p.9. Each regression analysis was indeed run twice, once with and once without including sociodemographic variables. Since both analyses yielded comparable results, in the main text we only reported the analyses where the sociodemographic variables were excluded. We have added the analyses including the sociodemographic variables in the Supplementary Materials (Tables 8-11). This is now specified at the beginning of the Results section on p. 15-16.*

*In addition to the regression analyses, we have now also checked the correlations between maternal depressive symptoms and the interaction behaviours considering the sociodemographic variables and observed a negative association between maternal depressive symptoms and MRIA (r=-.297, p=.047) and a positive association between maternal depressive symptoms and IN (r=.335, p=.024). Neither of these remained significant after controlling for multiple comparisons. These results have been added in the main manuscript on p. 18. Results are summarised in Table S9 in Supplementary Materials. We have now added a discussion of these findings on p.25-26.*

Study design:
4. The longitudinal study design allowed the authors to investigate the associations between mother-infant interaction characteristics at 9 months of age with maternal depression and infant temperament along the first year postpartum, and with child behavior at 3 years of age. It is important to consider the authors’ efforts to keep the sample intact over this time.
The authors mentioned that there were five points of assessment consisting of one pre- and four post-natal assessment points. However, they excluded the prenatal assessment point. I think it is important to include this assessment to avoid loss of the biological factors’ effect on the mother-infant interaction.

*Response: We would like to thank this reviewer for their thorough reading of our experimental procedure. We agree with the reviewer that collecting prenatal information is important. During the prenatal assessment in our study, we collected fetal heart rate and movements, but this data is still to be processed. We did not collect mothers’ depressive symptoms prenatally so unfortunately we cannot include this information in our manuscript. We have now included this information on p.8. We have also added a sentence in our limitations section on p.29: “Relatedly, collecting measures of prenatal maternal depressive symptoms as well as considering other (prenatal) biological factors will also be important in future studies.”*

5. The authors must specify the time of the four post-natal assessment points in the section of methods. These points appeared for the first time in Table S1 in supporting information file.

*Response: We are sorry for this omission. The exact times of the four post-natal assessment points are now specified on p.8.*

6. The major limitation in methods was the single point assessment of the main outcome variable (mother-infant interaction). It was assessed once at the age of 9 months. This main variable must be evaluated as the average of several assessments at several points of time as the maternal depression and infant temperament. In addition, it was unsuitable to depend on the undergraduate students in evaluation of the mother-infant interaction. Instead, expert developmental and behavioral pediatricians had to do this evaluation.

*Response: We agree with this reviewer that it is more difficult to draw conclusions from a single observation than a series of repeated observations. However, the single behavioural assessment at the age of 9 months was originally designed as main outcome measure of our infant longitudinal study and was carried out with great attention and care. We are therefore confident that it is valid and provides relevant information about child development and the mother-child relationship. We are currently conducting research where mother-infant interaction is observed longitudinally across multiple time points, but this was unfortunately not possible in the present study due to resource constraints and the fact that the protocol included several other key developmental constructs of interest. Specifically, our study also includes comprehensive longitudinal measures and tasks of attentional and social development, in addition to the measures of maternal depressive symptoms, mother-infants interaction and infant temperament development.
Regarding the coding of the mother-infant behaviours during interactions, we would like to assure this reviewer that the coders were fully and carefully trained by the first and second author following specific guidelines, and good interrater reliability was indeed achieved for most of the scales (p. 15). The research teams involved in carrying out this study have extended experience in working with undergraduate students and their guidance in behavioral coding use appropriate and rigorous training and data quality evaluation procedures.*

7. The Strengths and Difficulties Questionnaire (SDQ) should not be used for this young age, many available questionnaires could be used to investigate the child’s behaviors, emotions and relationships at the age of 36 months. (e.g. Child Behavior Checklist (CBCL), Ages & Stages Questionnaire: Social-Emotional (ASQ: SE)

*Response: We acknowledge this reviewer’s view that there are other tools that can be used to investigate children’s behaviours at 3 years of age. However, while the Strengths and Difficulties Questionnaire (SDQ) was originally designed to assess behaviours, emotions and relationships in children and young people aged 4–17 years, it has previously been successfully used with parents (or educators) of 2–4-year-olds – as well as with youths aged 18 and over. In line with this, Goodman (2001) refers to the SDQ as a brief measure of prosocial behavior and psychopathology of 3–16-year-olds that can be completed by parents, teachers, or youths. There have also been previous studies which used the SDQ with younger children (e.g., Du et al., 2008).
We would furthermore like to draw this reviewer’s attention to a study by Goodman and Scott (1999) who directly compared the SDQ and the CBCL. They found that scores from the SDQ and CBCL were highly correlated and equally able to discriminate psychiatric cases from controls. They also report that the SDQ was significantly better than the CBCL at detecting inattention and hyperactivity, and at least as good at detecting internalizing and externalizing problems. Also, mothers of low-risk children were twice as likely to prefer the SDQ. Taken together, we are confident that the SDQ is a valid and appropriate measure for our study.*

References:

Du, Y., Kou, J., & Coghill, D. (2008). The validity, reliability and normative scores of the parent, teacher and self report versions of the Strengths and Difficulties Questionnaire in China. *Child and adolescent psychiatry and mental health*, *2*(1), 1-15.

Goodman, R. (2001). Psychometric properties of the strengths and difficulties questionnaire. *Journal of the American Academy of Child & Adolescent Psychiatry*, *40*(11), 1337-1345

Goodman, R., & Scott, S. (1999). Comparing the Strengths and Difficulties Questionnaire and the Child Behavior Checklist: is small beautiful?. *Journal of abnormal child psychology*, *27*, 17-24.

8. Confounding variables were not considered in this study, which might affect mother-infant relationship as health status of the infant or the mother, socioeconomic variables, social support, work status, and marital status.

*Response: As mentioned in our response to point 3 above, these variables were collected and considered in our preliminary analyses, and are now also added in the Supplementary Materials (Supp Tables 9-11). We have now specified this at the beginning of the Results section on p. 15-16, and added a discussion on the impact of SES in light of the associations with maternal depressive symptoms on p. 25-26.*

Results:

Were well presented.
9. Table S6 in the supporting file: It described the associations between infant Falling Reactivity at each time point and Dyadic Affective Mutuality observed at 9 months. These associations were reported to be positive. Thus, authors have to revise for the negative sign in each correlation.

*Response: We thank this reviewer for spotting this mistake. It has now been corrected.*

Discussion:
10. The finding of a lack of association between interaction behaviors and maternal depressive symptoms was not acceptable. This may be due to the dependence on single evaluation of interaction behavior as mentioned before; or because all participants had minimal depression symptoms that could not impact the interaction behavior. The other two findings are acceptable.

*Response: We agree with this reviewer that a variety of factors might have precluded us from detecting a significant relation between maternal depressive symptoms and the child, mother, and mother-child interaction behaviours coded in this study. We now report and discuss in our manuscript that when we include SES factors in the analyses we find some correlations, though these do not survive multiple comparison correction. We addressed the point about a single evaluation point for the interaction in Point 6 above. Although our sample was not a clinical sample, the mean BDI-II score was 8.4 with a standard deviation of 6.7, clearly indicating that scores were not at floor and showed plenty of variation that maternal depressive symptoms could potentially associate with. We provide in-depth interpretations of both the significant and non-significant associations within the context of the existing literature in the Discussion on p. 24-25.*

Reviewer #2: this study is very interesting for readers and caregivers, all the sections are very well described and data were collected methodologically correctly.
those aspects were very well descibed in the methods and result section

*Response: We thank this reviewer for their overall positive comments on our manuscript.*
