# Supplementary material for: Mother-infant interaction characteristics associate with infant falling reactivity and child peer problems at pre-school age
Source: PLoS One. 2024 Jun 4;19(6):e0302661. doi: 10.1371/journal.pone.0302661 (PMC11149888; doi:10.1371/journal.pone.0302661)
Supplement: Attachment — Submitted filename: Reviewer 1 comments 2.docx [file pone.0302661.s004.docx]

**Reviewer 1 comments 2-4-2024:**

I’m pleased to review the updated version of this interested manuscript “Mother-infant interaction characteristics associate with infant falling reactivity and child peer problems at pre-school age”. PONE-D-23-29119R1

I would like to thank the authors as all previous comments have been addressed.

The new added paragraphs and explanation have enriched the manuscript.

I think this updated version is accepted and ready for publication
