# Supplementary material for: Mother-infant interaction characteristics associate with infant falling reactivity and child peer problems at pre-school age
Source: PLoS One. 2024 Jun 4;19(6):e0302661. doi: 10.1371/journal.pone.0302661 (PMC11149888; doi:10.1371/journal.pone.0302661)
Supplement: Attachment — Submitted filename: Response to Reviewers_R2.docx [file pone.0302661.s005.docx]

PONE-D-23-29119R1

Mother-infant interaction characteristics associate with infant falling reactivity and child peer problems at pre-school age

PLOS ONE

**Response to reviewers**

Please find our answers in *italics* below each reviewer’s comment.

Reviewer #3:

Thank you for the opportunity to review the study entitled 'Mother-infant interaction characteristics associate with infant falling reactivity and child peer problems at pre-school age.' This study is very interesting and well-organized, addressing a topic that needs to emerge more in the literature. Although I have noticed that the authors have extensively revised the paper, significantly improving their manuscript, I believe it is important for the authors to further explore the developmental age they are addressing. Specifically, while the literature regarding mother-infant exchanges has been thoroughly explored, it would be important for the authors to also delve deeper into the changes that occur as the child grows. With the acquisition of greater autonomy and what M. Mahler has defined as a period of separation-individuation in the mother-child dyad, the child gains a greater sense of agency, which will continue to evolve during toddlerhood, the developmental phase addressed by the authors. It is important that this be better defined in the introduction and that the results be discussed based on the specific developmental context of the child and the dyad as a whole. Below, I highlight some literature that could be useful:
- Ballarotto (2023). Parental sensitivity to toddler's need for autonomy: An empirical study on mother-toddler and father-toddler interactions during feeding and play. Infant Behavior and Development
- Kemppinen (2006). The continuity of maternal sensitivity from infancy to toddler age. Journal of Reproductive and Infant Psychology
- Lecuyer-Maus (2000). Maternal sensitivity and responsiveness, limit-setting style, and relationship history in the transition to toddlerhood. Issues in Comprehensive Pediatric Nursing
- Ballarotto (2021). Mother-child interactions during feeding: A study on maternal sensitivity in dyads with underweight and normal weight toddlers. Appetite
- Andreadakis (2020). Toddler temperament, parent stress, and autonomy support. Journal of Child and Family Studies

*We are grateful to the Reviewer for their comments on our work and for pointing out the importance of highlighting the transition to toddlerhood with particular attention to the acquisition of greater autonomy. We have read the suggested references with interest and included those in the manuscript in the Introduction and in the Discussion when referring to our investigation of social and emotional outcomes at 3 years of age. Specifically, in the introduction we have added the references by Kemppinen et al. (2006) [37] and Lecuyer-Maus (2000) [43] and on p. 6 we have added the sentence:*

*“More generally, and in accordance with a transactional perspective, during the transition to toddlerhood, mother-infant interactions lay the foundation for future developmental outcomes (e.g. [43]).”*

*In the Discussion we have expanded on the parents’ autonomy support findings citing Ballarotto et al. (2023) [81], Andreadakis et al. (2020) [87], and Joussemet et al. (2008) [80]. On p. 24 we have added:*

*“Autonomy is considered a fundamental child need and parental support for autonomy is arguably one of the most important aspects of parenting for the development of child independence [80]. Requests for autonomy are often found among the most frequent cues in toddlers’ interactions with both their mother and father (e.g. [81]), highlighting the emerging need for autonomy in the transition from infancy to toddlerhood.”*

*Later on in the paragraph we have added:*

*“In addition, another recent study [87] reported differential patterns of associations between toddler temperament, parental stress and support for autonomy depending on the temperamental characteristics. Parent stress was found to fully account for the negative association between toddler negative affectivity and parent autonomy support, suggesting that toddler temperament affects parent wellbeing, which in turn is related to parenting practices.”*

Furthermore, in the study limitations, it is important to highlight the constraints of certain self-reports (such as the SDQ, particularly in situations of parental stress). You can refer to the study by: Trumello (2023) Mothers and fathers of pre-school children: a study on parenting stress and child’s emotional-behavioral difficulties

*We thank the reviewer for this additional point and reference. We have added this limitation on p. 25:*

*“Collecting information from additional observers of the child (e.g., other family members, educators, etc.) would have allowed a more objective assessment of the infant temperament as well as the child’s social and emotional outcome at 3 years of age.”*
